# Pleomorphic adenoma of the nasal septum: a case report

**DOI:** 10.1186/1752-1947-2-349

**Published:** 2008-11-17

**Authors:** Polycarp Gana, Liam Masterson

**Affiliations:** 1 ENT Department, Queen Alexandra Hospital, Portsmouth, PO6 3LY, UK; 2 ENT Department, Edith Cavell Hospital, Bretton Gate, Peterborough, PE3 9GZ, UK

## Abstract

**Introduction:**

Pleomorphic adenomas are the most common benign tumour of the major salivary glands. In addition, they may also occur in the minor salivary glands of the hard and soft palate. Intranasal pleomorphic adenomas are unusual and may be misdiagnosed because they have greater myoepithelial cellularity and fewer myxoid stromata compared to those elsewhere.

**Case presentation:**

We present the case of a 61-year-old man who presented with a 2-year history of left nasal obstruction, occasional epistaxis and facial pain. Radiological examination demonstrated well pneumatised paranasal sinuses and a soft tissue mass in the anterior aspect of the left nasal cavity. In this patient, an intranasal approach was used to achieve a wide local resection.

**Conclusion:**

Pleomorphic adenomas are rare tumours of the nasal cavity and have been shown to be misdiagnosed in over half of cases leading to more aggressive treatment than is necessary. If unilateral nasal obstruction is the main presenting complaint, we suggest consideration of this diagnosis. In view of the potential for tumour recurrence, long-term follow-up and careful examination of the nose with an endoscope are necessary.

## Introduction

Salivary gland tumours constitute about 3% [1] of all neoplasms. The majority of these tumours are benign and about 70% are pleomorphic adenomas. A small minority (8%) are located in the oral cavity, neck and nasal cavity. We present a rare case of pleomorphic adenoma of the nasal septum.

Several benign lesions of the septum such as leiomyoma, osteochondroma and transitional cell papilloma have been reported in literature. The other differential diagnoses may include malignant tumours such as melanoma, adenoid cystic carcinoma and squamous cell carcinoma. The majority of these tumours arise from the mucosa of the bony and cartilaginous septum.

Nasoseptal swell body is a discrete area of erectile tissue in the submucosa over the anterior nasal septum. In some individuals, it can present as a suspicious lesion. It does not have a significant relevance when considering the differential diagnosis in this patient given the enormous size of the septal mass. However, in smaller septal swellings, it could be given consideration.

## Case presentation

A 61-year-old man presented with a 2-year history of left nasal obstruction, occasional epistaxis and facial pain. There was no history of visual defect, atopy or previous trauma to the nose. His weight was stable and his general health was satisfactory.

Rigid endoscopy of the nose revealed a grossly deviated septum to the right and a large polypoid mass filling the left nasal cavity. There was no evidence of rhino-sinusitis and his postnasal space was normal. There were no palpable neck nodes.

**Figure 1:**
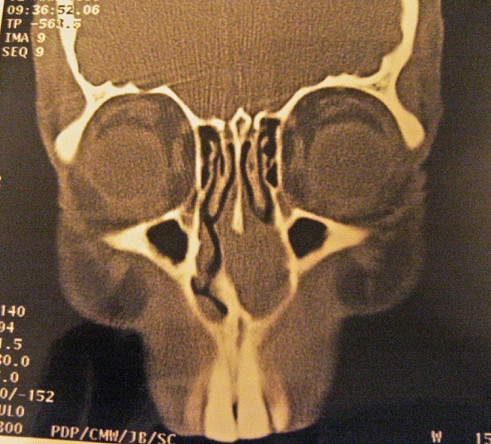
Sinus computed tomography scan (coronal section) showing a 2 × 2.2 × 1.4 cm mass in the left nasal cavity.

Radiological examination (CT scan) demonstrated well pneumatised para-nasal sinuses and a soft tissue mass in the anterior aspect of the left nasal cavity. This was located anterior to the inferior turbinate and arising from the septum. The smooth surface, preservation of mucosal lining and the localised nature of the mass were consistent with a benign lesion (Figure 1).

In this patient, pre-operative incisional biopsy of a smooth, rounded and firm mass arising from the septal mucosa established the diagnosis of a pleomorphic adenoma. A submucous resection was used as an approach to the tumour and as a method of excising the mass with the segment of septal cartilage attached to it. This was deemed necessary during surgery due to evidence of partial thinning of the septal cartilage adjacent to the lesion. A 1 cm margin of normal ipsilateral mucosa and the surrounding perichondrium were also excised. The septal mucosa of the opposite side was preserved.

Histological analysis of the tumour confirmed a benign pleomorphic adenoma with no focus of malignant change; the resection margins were clear. The patient was discharged on the same day, and the postoperative course was uneventful. After 4 years, the patient had experienced no further problems with the nasal airway, and repeated nasal endoscopic examination revealed no recurrence of the disease.

## Discussion

The most common tumours of the major salivary glands are pleomorphic adenomas, but in rare instances, they can occur in the respiratory tract (via minor salivary glands). Cases have been reported in the nasal cavity, paranasal sinuses, nasopharynx, oropharynx, hypopharynx, and larynx. In the upper respiratory tract, the most favoured site of origin is the nasal cavity, followed by the maxillary sinus and the nasopharynx [2]. The first reported case in the literature of a pleomorphic adenoma of the nasal cavity was in 1929 [3]. Although the vast majority of minor mucous and serous glands are located in the lateral nasal wall, pleomorphic adenomas in the nasal cavity mostly originate from the nasal septum. Larger studies of intranasal pleomorphic adenoma include 40 cases reported by Compagno and Wong and 59 cases reported by Wakami *et al. *[4,5].

The majority of tumours present between the age of 30 and 60 years and are slightly more common in women. Typical presenting features include unilateral nasal obstruction (71%) and epistaxis (56%). Other signs and symptoms include a mass in the nose, nasal swelling, epiphora, and mucopurulent rhinorrhoea [4].

**Figure 2:**
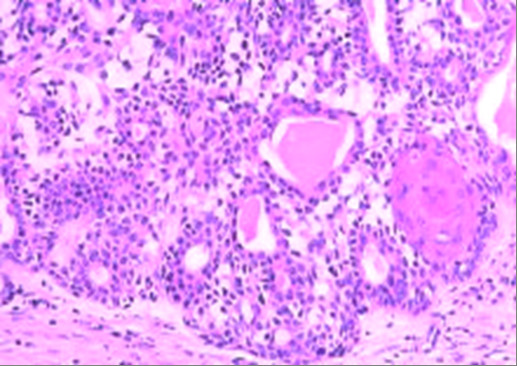
Histology section demonstrating a minor salivary gland pleomorphic adenoma with increased myoepithelial cellularity and a relatively small stromal component.

Pleomorphic adenomas are characterised by epithelial tissue mixed with tissues of myxoid, mucoid or chondroid appearance. Histologically, pleomorphic adenoma of the aerodigestive tract may resemble aggressive epithelial tumours because of the high cellularity and lack of a stromal component (Figure 2). Importantly, this feature is not in keeping with that of the major salivary glands which demonstrate relatively reduced myoepithelial cellularity. Occasionally, pleomorphic adenomas are composed almost entirely of epithelial cells with few or no stromata. This can lead to misdiagnosis as a carcinoma. A fact reflected by Compagno and Wong wherein 55% of cases were initially not accurate [4].

Wide local resection with histological clear margin is generally agreed as the treatment of choice for benign salivary gland tumours. Postoperative radiotherapy has been advocated by some authors in circumstances where residual disease was apparent [6]. In the case of intranasal pleomorphic adenoma, several surgical approaches have been used to achieve wide local clearance and these include intranasal, transnasal endoscopic, external rhinoplasty, lateral rhinotomy and mid facial degloving [7].

In their reported series of 40 patients, Compagno and Wong used the lateral rhinotomy approach for excision of tumour in the majority of the patients. Only three patients had a recurrence of disease after 3 years of follow-up. The recurrent lesions constituted more stroma than cellular elements and the former is thought to provide the focus for recurrence [4].

The outlook for intranasal mixed tumours is better than for those in other ectopic sites, because they show early symptoms leading to an early diagnosis. Involvement of the surrounding structures such as bone is rare since the tumours have sufficient space to expand within the nasal cavity [7].

A neoplasm originating from the nasal septum has a higher risk of malignancy compared to other sites in the nose [8]. Occasionally, pleomorphic adenoma can behave in a malignant fashion, the most common variant being carcinoma ex pleomorphic adenoma which has a potential to metastasise. The predominant metastatic site is bone but spread to lungs, regional lymph nodes and liver has been documented [9]. Ten cases of metastasising pleomorphic adenoma of the parotid gland and three patients with metastatic pleomorphic adenoma of the minor salivary glands have been reported in the literature [10].

## Conclusion

In summary, pleomorphic adenomas are rare tumours of the nasal cavity. They have a higher epithelial and lower stromal component compared to their major salivary gland counterparts and may be misdiagnosed at an early stage leading to more aggressive treatment. We suggest consideration of this diagnosis if the patient has unilateral nasal obstruction or epistaxis as a presenting complaint. In view of the potential for tumour recurrence, long-term follow-up and careful examination of the nose with an endoscope are necessary.

## Abbreviations

CT: computed tomography.

## Consent

Written informed consent was obtained from the patient for publication of this case report and any accompanying images. A copy of the written consent is available for review by the Editor-in-Chief of this journal.

## Competing interests

The authors declare that they have no competing interests.

## Authors' contributions

PG and LM both contributed to conception and design, and carried out the literature research, manuscript preparation and manuscript review. Both authors read and approved the final manuscript.
